# Hysteroscopic Removal of an Intrauterine Contraceptive Device Obstructed by Polyps: A Case Study

**DOI:** 10.7759/cureus.77990

**Published:** 2025-01-26

**Authors:** Aishwarya Gupta, Sandhya Pajai, Lucky Srivani Reddy, Sachin Rathod

**Affiliations:** 1 Department of Obstetrics and Gynaecology, Datta Meghe Institute of Higher Education and Research, Wardha, IND

**Keywords:** endometrial polyp, hysteroscopy, intrauterine contraceptive device (iud), iud obstruction, minimally invasive surgery

## Abstract

In this case report, a patient presented with an intrauterine contraceptive device (IUD) obstruction caused by the presence of an endometrial polyp. Despite IUDs being known for their effectiveness as long-term contraceptives, it is important to note that intrauterine abnormalities can compromise their functionality. The obstruction was successfully diagnosed and treated using hysteroscopy, a minimally invasive procedure that enables direct visualization and removal of the polyp. This case highlights the important role of hysteroscopy in managing the complications associated with intrauterine devices.

## Introduction

Amongst the most effective forms of long-acting and reversible contraception are intrauterine contraceptive devices (IUDs). They are favored due to their high efficiency, low maintenance, and long-term effects [1]. However, despite being widely used and generally safe, complications may arise. The complications include device expulsion, malpositioning, and perforation, as well as obstruction from pathology within the uterus, e.g., fibroids or polyps.

Benign endometrial polyps are unusual growths on the inner lining that can cause symptoms such as irregular bleeding, menorrhagia, and infertility. Polyps are often asymptomatic and identified incidentally through imaging or hysteroscopy procedures [2]. However, if symptomatic, they can significantly compromise a patient’s quality of life and reproductive health. The presence of an endometrial polyp may obstruct the IUD, leading to symptoms that require additional intervention.

The management of intrauterine pathology has been revolutionized by hysteroscopy. This minimally invasive technique provides direct visualization of the uterine cavity, allowing diagnosis and treatment in a single procedure [3]. It provides a precise method for the removal of intrauterine lesions with minimal patient discomfort, thereby facilitating expedited patient recovery. This compelling case report highlights the successful hysteroscopic removal of an IUD obstructed by an endometrial polyp, demonstrating the importance of prompt and effective intervention in such cases.

## Case presentation

The patient was a 35-year-old, para one living one with no previous medical history, who complained of irregular bleeding and lower abdominal pain. She had a copper IUD placement for contraception three years ago with no adverse early experiences. Recently, she mentioned experiencing a missed period, along with both heavy and occasional light bleeding, and also experiencing blotchy, sporadic lower abdominal pain over the past six months.

**Figure 1:**
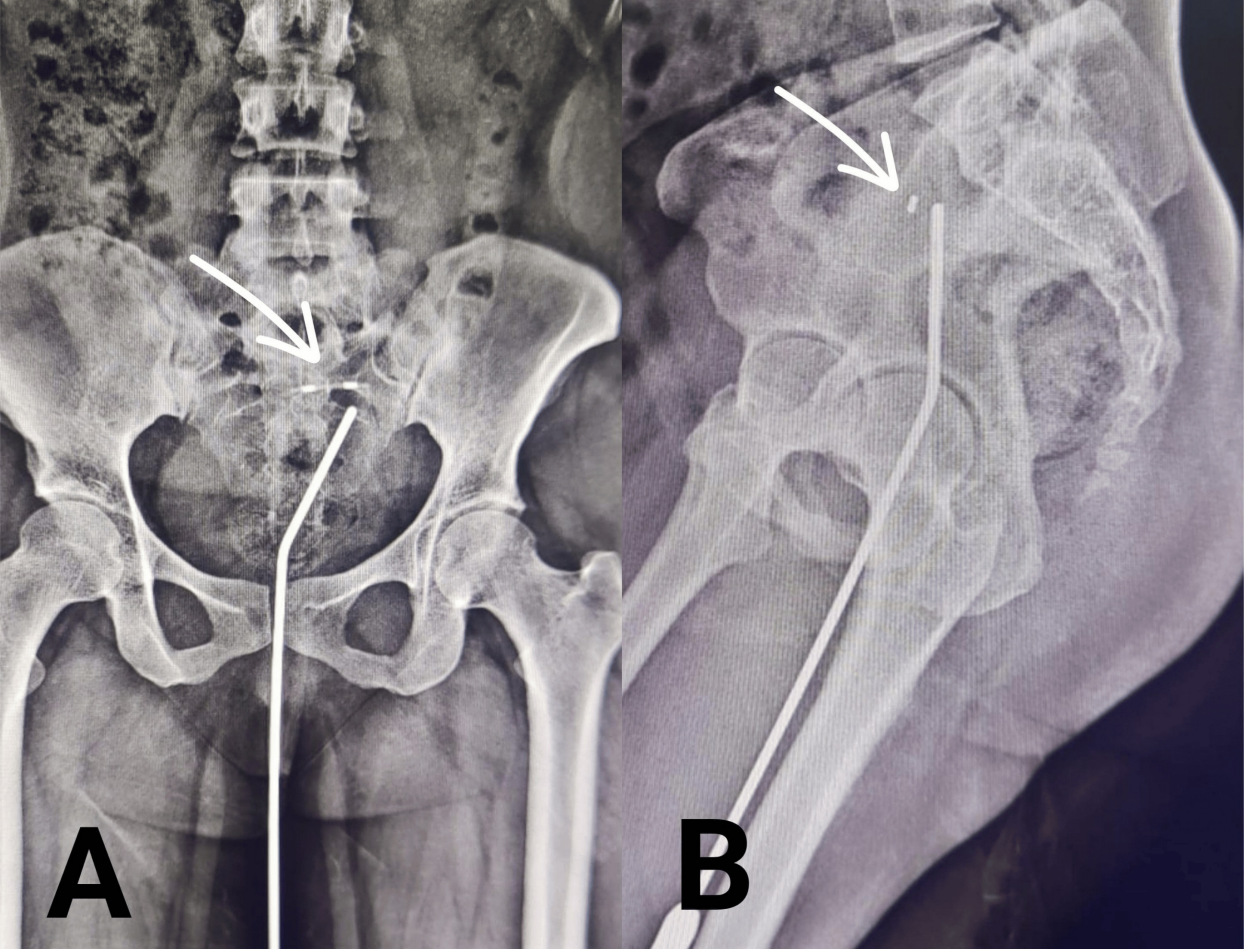
X-ray of the pelvis with uterine sound in situ. (A) Anteroposterior view and (B) lateral view. The location of the intrauterine contraceptive device is marked with white arrows.

During the initial pelvic examination, it was observed that the IUD strings were not visible at the cervical os, raising concerns about the device's positioning. The patient also disclosed that she had visited a local healthcare provider who attempted to remove the IUD, but only managed to remove a portion of it (vertical arm along with thread). An X-ray with uterine sound in situ was carried out to verify the intrauterine placement of the IUD, and it was confirmed to be properly positioned, as illustrated in Figure 1.

**Figure 2:**
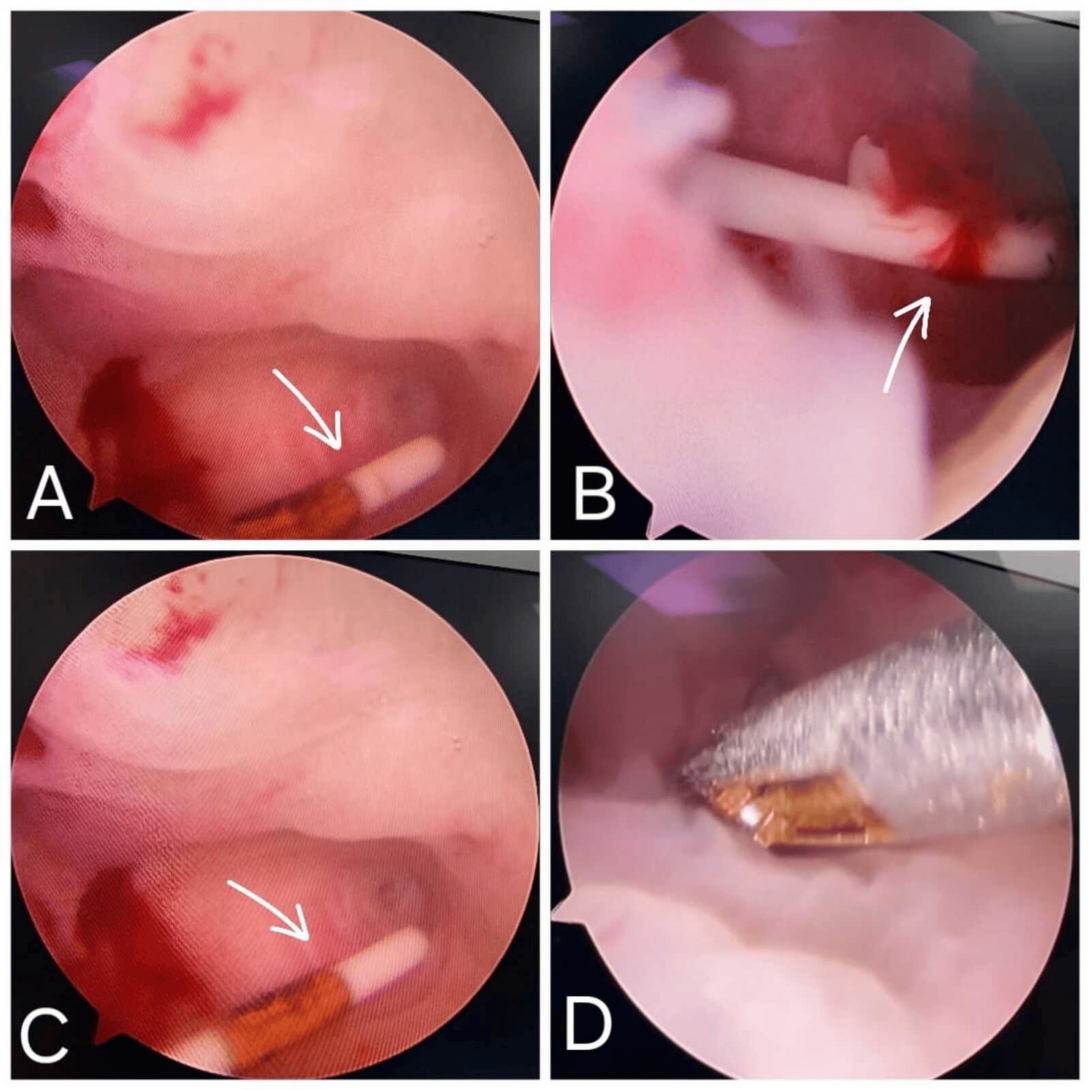
Hysteroscopic images showing a small polyp obscuring the intrauterine contraceptive device (IUD) as seen in images A, B, and C, and the use of hysteroscopic scissors for resection (D). The IUD is marked with a white arrow.

**Figure 3:**
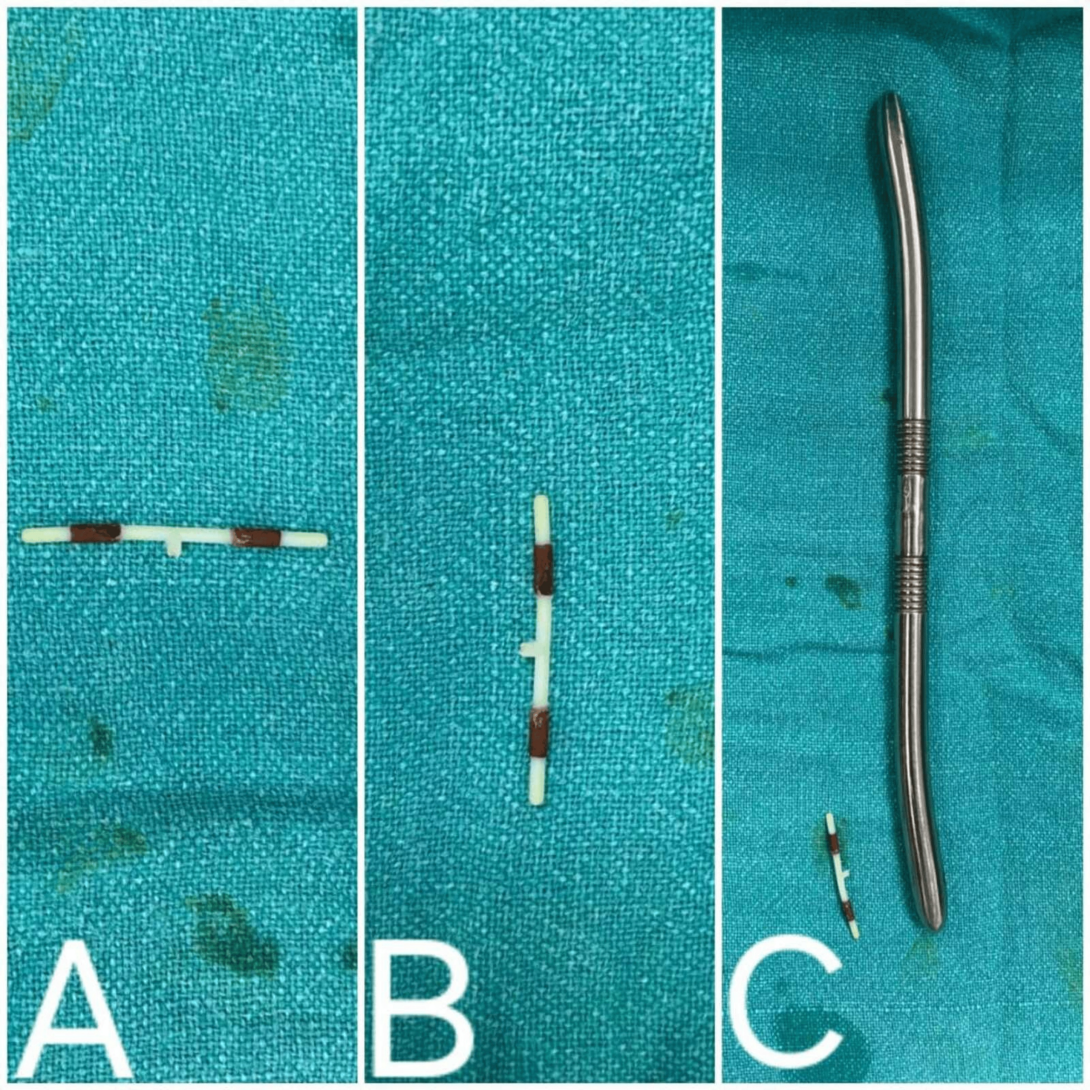
Intrauterine contraceptive device post resection. (A) Horizontal view, (B) vertical view, and (C) size seen next to a Hegar dilator.

The patient was advised of the need for hysteroscopic IUD removal and consented to the procedure. The patient was taken to the operating room and was induced with general anesthesia. An operative hysteroscope was gently placed in the uterine cavity where the interior of the uterus can then be observed. On examination, a small round polyp was noted, which was overlying the IUD as shown in Figure 2. With the help of hysteroscopic scissors besides bipolar electrosurgery, the polyp was shaved carefully. The bipolar electrosurgery gave the possibility to operate selective coagulation and cutting, thus, excluding intraoperative bleeding. After the complete resection of a polyp, the IUD was gently grasped hysteroscopically using forceps and pulled out from the uterus as shown in Figure 3. The entire procedure took about 30 minutes.

Uterine cavity assessment was unremarkable and revealed no other pathology during surgery and the operation was successfully done. After this, the patient was observed in the postoperative recovery room for four hours for signs of early complication, including bleeding and or excessive pain. She was then discharged on the same day under the advice of complete rest for some days and no rigorous activities.

The patient was reviewed at the six-week follow-up, which showed that the patient was symptom-free. She did not report any abnormal bleeding.

## Discussion

The case also points out the extent of considering endometrial polyps as one of the causes of IUD obstruction. While it is noteworthy that IUDs, in general, are safe and effective methods of contraceptives, intrauterine pathology complicates the use of IUDs. Although most endometrial polyps are not malignant, they can often result in severe pain and inconvenient positioning and operation of an IUD [1,4].

IUD-related complications are better solved by hysteroscopy. This is so because with it one can have a direct view of the uterine cavity to be able to identify and therefore remove pathology that is at the root of the complete resolution of symptoms [5]. In this case, a woman’s IUD was dislodged due to the presence of an obstructing polyp; with a hysteroscopic removal of this polyp, the IUD was successfully retrieved and the patient’s symptoms resolved [6].

Another benefit that is linked to hysteroscopic removal is its minimally invasive approach, meaning that recovery time and risks of complications will be minimal. The technique affords fewer chances of blind operations causing uterine perforation and equally guarantees optimal elimination of intrauterine pathology [7]. This is even so as it concerns an IUD because if the device is incomplete or damaged then there will be even more complications.

Moreover, although those risks are low if managed by an expert surgeon, the same cannot be said for all patients. This patient had no complications and her postoperative course was smooth [5]. This case also stresses the importance of careful examination and appropriate treatment interventions regarding potential IUD-related complications. It also depicts the ability of hysteroscopy in the modern practice of gynecology as one of the safest as well as efficient methods to address intrauterine pathologies [8]. The various advantages and potential complications are enumerated below in Table 1.

**Table 1:** Advantages and potential complications of hysteroscopic removal.

Advantages of hysteroscopic removal	Potential complications
Minimally invasive with a quick recovery time	Bleeding
Direct visualization ensures complete polyp removal	Infection
Reduced risk of uterine perforation compared to blind removal techniques	Uterine perforation (rare)

## Conclusions

IUD blockages resulting from the presence of endometrial polyps can be safely and effectively resolved through the use of hysteroscopic surgery, a minimally invasive procedure. This specific instance underscores the essential role of employing advanced minimally invasive techniques in modern gynecological care.
